# Notes on the Cave Committee's Report

**Published:** 1921-06-18

**Authors:** 


### Notes on the Cave Committee's Report.

One of the most important of the Committee's recommendations is the setting up of a Hospitals Commission, which will be a body formed on the lines of the University Grants Commission, to be appointed by the Ministry of Health and to consist of not less than twelve members, of whom the chairman and four others would be selected by the Minister of Health himself, and one by the Secretary for Scotland, and of the remainder one each by the following bodies, namely: The Joint Committee of the Red Cross Society and the Order of St. John of Jerusalem; King Edward's Hospital Fund for London; the British Hospitals Association; the Royal College of Physicians; the Royal College of Surgeons; the British Medical Association; and the Scottish Committee of the British Medical Association. Service on the Hospitals Commission should be voluntary, but it would be advised by an expert and will require a small clerical staff. The expense would fall on the votes of the Ministry of Health.

The Hospitals Commission would, however, deal only with larger propositions, such as the final voice in the distribution of grants and major questions of finance and policy. Other affairs, which would lie between the minute detail of the administration of individual hospitals and the large matters decided by the Hospitals Commission, would be the business of local Voluntary Hospitals Committees. In this connection the organisation for London is proposed to be King Edward's Hospital Fund, and throughout England and Wales normally the unit suggested should be the county or county boroughs, in which a Voluntary Hospital Committee should be nominated by the Lord Lieutenant and approved by the Hospitals Commission. The Committee would have no compulsory powers, and would derive authority from their personnel and their functions in relation to the grants they would recommend. It is thought that the local Committee could do much to adjust the anomaly of vacant beds in one part of the district and a heavy waiting-list in another, to which the attention of the Cave Committee was called by several important witnesses. Although no definite recommendation was made, the system of the larger hospitals linked up with smaller hospitals is evidently favoured by the Committee.

It is noticed that the report refers to "the cost per occupied bed" as being unsatisfactory, as it is so largely affected by the design and internal arrangements of a hospital and other conditions. The Committee, however, clearly insist upon the enforcement of the Uniform System of Accounts, and refer to the difficulty experienced by the inquiry through the use of varying systems in the Provinces. Having through the Local Committees brought about the general use of the Uniform System, it would then be much simpler to check expenditure and to bring about economies.

Another interesting point is that the Committee of Inquiry hold that the Approved Societies are not under any obligation, legal or equitable, to provide the whole cost of the maintenance and treatment of their members in hospital, although it is strongly felt that they do owe to the hospitals a large measure of support. The Committee hope that the action of the National Deposit Friendly Society, one of the largest of the Societies, in applying one-third of its available surplus for the benefit of the hospital and nursing services, will be followed by others. Reference is made to the remarkable fact that most of the companies insuring against claims for workmen's compensation, although profiting largely by the treatment of insured workmen in hospitals, make little or no contribution to hospital funds.

No hospital should be without a copy of this excellent report, which can be obtained from H.M. Stationery Office, Imperial House, Kingsway, W.C. 2. The price is 4d. net.

